# Genome Sequence of Mycobacterium Phage LilHazelnut

**DOI:** 10.1128/MRA.00431-19

**Published:** 2019-05-09

**Authors:** Ryan A. Shanks, Ashley N. Hazel, William H. Jones, Miriam Segura-Totten

**Affiliations:** a Department of Biology, University of North Georgia, Dahlonega, Georgia, USA; DOE Joint Genome Institute

## Abstract

Here, we describe LilHazelnut, a novel mycobacteriophage that infects Mycobacterium smegmatis mc^2^155. LilHazelnut is a cluster Q phage that shares 99% nucleotide identity with phage Giles, is 53,746 bp in length, and has a G+C content of 67.5%. LilHazelnut is a temperate *Siphoviridae* virus, as is typical of cluster Q family members.

## Abstract (precis)

Here, we describe LilHazelnut, a novel mycobacteriophage that infects Mycobacterium smegmatis mc^2^155. LilHazelnut is a cluster Q phage that shares 99% nucleotide identity with phage Giles, is 53,746 bp in length, and has a G+C content of 67.5%.

## ANNOUNCEMENT

The increasing number of isolated and characterized bacteriophage provides a valuable database of genetic diversity allowing for the identification of both shared and novel gene products between bacteriophage species (1). Here, we describe a new mycobacteriophage, LilHazelnut, isolated as part of the Science Education Alliance Phage Hunters Advancing Genomics and Evolutionary Science (SEA-PHAGES) program (2) from a single plaque in an enrichment of a 0.22-μm filtered 7H9 liquid medium-washed soil sample from North Georgia (coordinates 24.527904, 83.987719) using the host Mycobacterium smegmatis mc^2^155 and purified using three consecutive serial dilutions. LilHazelnut, a *Siphoviridae* virus, has an icosahedral head and a 195-nm tail, and it produces clear round plaques of 0.3 mm with hazy edges.

Genomic DNA was isolated from LilHazelnut lysates with a Wizard DNA extraction kit (Promega). A NEBNext Ultra II FS kit with dual-indexed barcoding was used to prepare a sequencing library from genomic DNA. This library plus those from 47 other phages were pooled and run on an Illumina MiSeq instrument. This assay yielded ∼895,000 single-end 150-base reads from the LilHazelnut library, and when assembled, these reads provided ∼2,384-fold coverage of the LilHazelnut genome. These raw reads were assembled using Newbler v2.9 with default settings. The resulting single phage contig was checked for completeness, accuracy, and phage genomic termini using Consed v29 as previously described (3). LilHazelnut contains a circularly permuted genome consisting of 53,746 bp with a G+C content of 67.5%.

The LilHazelnut genome was annotated using Glimmer v3.02 (4), GeneMark v2.5p (5), BLASTP v2.7.1 (https://blast.ncbi.nlm.nih.gov/), HHPred v3.0beta (6), and Phamerator (7). The E value cutoff used for BLASTP and HHPred was 10 e−4. Automatic genome annotation followed by manual refinement revealed that LilHazelnut is part of the cluster Q phages and is predicted to have no tRNAs/transfer-messenger RNA (tmRNA) genes but has 85 protein-coding genes and 35 genes with assigned functions. Functional assignments for predicted proteins included virion structure and assembly proteins, as well as terminase, integrase, excise, and lysin A and B proteins. Except for genes 2 to 4, both ends of the phage genome are transcribed in the forward direction, while the genes in the middle portion of the genome are transcribed in the reverse direction.

LilHazelnut displays genome-wide similarity to the other 11 temperate phages in this cluster, sharing 99.99% nucleotide identity with its closest relative Giles (GenBank accession no. NC_009993). Identity to Giles was determined through a nucleotide BLAST search (https://blast.ncbi.nlm.nih.gov/Blast.cgi). Genes *gp29* and *gp30*, which form part of the integration cassette in LilHazelnut, are conserved in phages Kinbote (GenBank accession no. KT22940) and Giles. The attachment site (*attP*) in LilHazelnut is identical to that of Giles, and is also found upstream of the integrase gene (positions 25134 to 25179). Similarly, *gp47*, which in Giles functions as a phage repressor, is conserved in LilHazelnut as *gp49*. A putative DNA binding domain in this repressor is conserved across all cluster Q members (8).

### Data availability.

LilHazelnut is available at GenBank with accession no. MF919517. Sequencing reads are part of the Sequence Read Archive with SRA accession no. SRX5282798 under BioProject accession no. PRJNA488469.
